# A Rare Presentation of Nodular Amyloidosis on the Lower Back

**DOI:** 10.7759/cureus.5864

**Published:** 2019-10-08

**Authors:** Yelena Dokic, Paul Subrt, Jaime Tschen

**Affiliations:** 1 Dermatology, Baylor College of Medicine, Houston, USA; 2 Dermatology, Katy Westside Dermatology, Houston, USA; 3 Dermatology, St. Joseph Dermatopathology, Houston, USA

**Keywords:** primary localized cutaneous amyloidosis, nodular amyloidosis, lichen amyloidosis, papular amyloidosis

## Abstract

Primary localized cutaneous amyloidosis (PLCA) occurs when amyloid is deposited only within the skin and there is no evidence of systemic involvement. Nodular amyloidosis is the rarest subtype of PLCA. It typically involves the acral regions but can sometimes present on the head and neck. The condition usually presents clinically as a single tan or yellow nodule or plaque that may appear waxy. Herein, we present a rare case of a 66-year-old man with nodular amyloidosis on the lower back.

## Introduction

Amyloidosis involves the deposition of amyloid protein into various tissues of the body. Primary localized cutaneous amyloidosis (PLCA) occurs when amyloid is deposited only within the skin, and there is no evidence of systemic involvement. Three subcategories exist within the PLCA category: macular, papular, and nodular amyloidosis. Nodular amyloidosis, or nodular localized cutaneous amyloidosis, is the rarest subtype of PLCA. Herein, we present a rare case of a 66-year-old man with nodular amyloidosis on the lower back, which had been present for several months at the time of presentation.

## Case presentation

**Figure 1:**
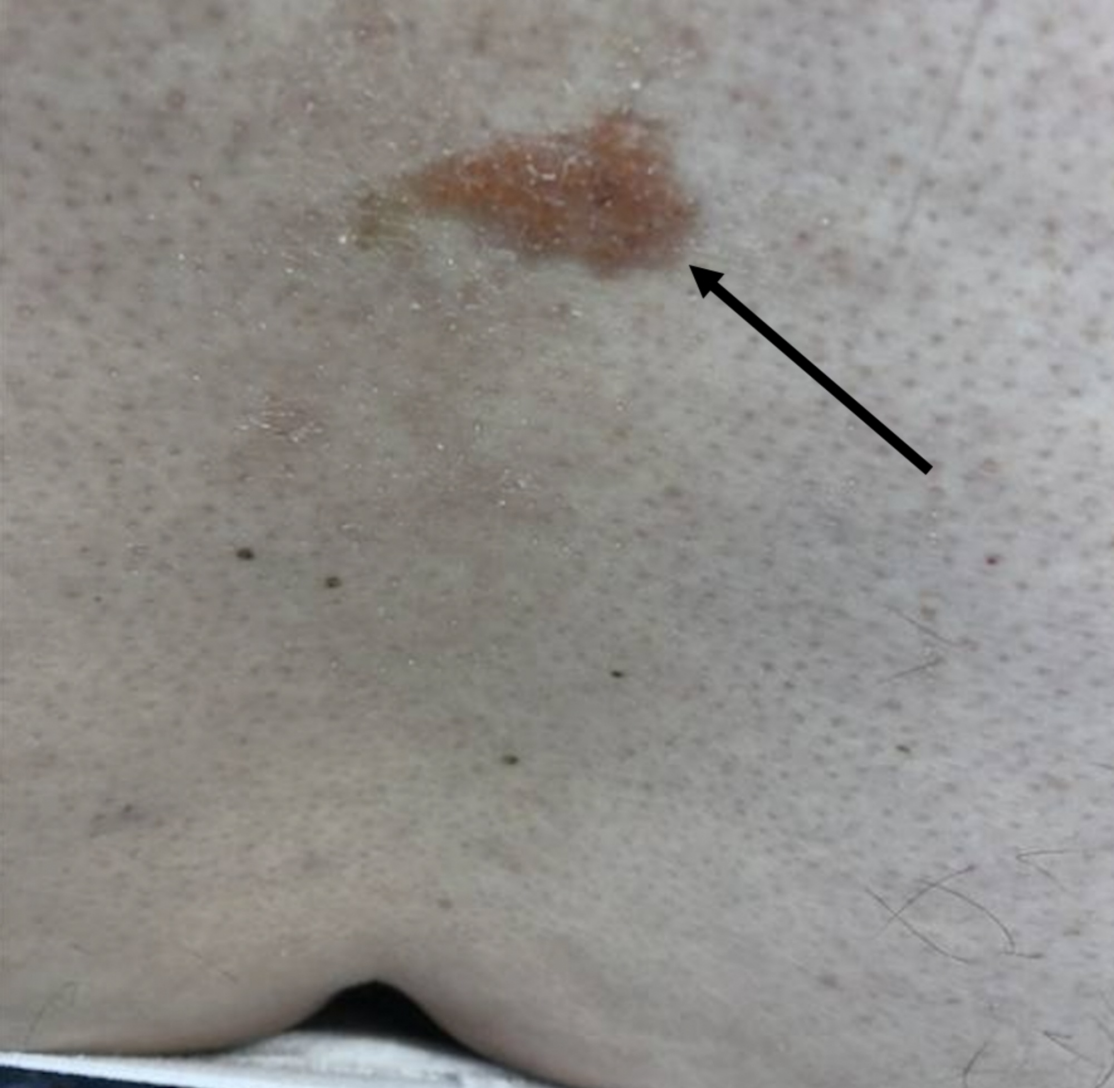
A yellowish-red plaque on the lower back. Arrow indicates plaque, 2 cm by 1 cm in size.

A 66-year-old man presented with a yellowish, red plaque (2 cm by 1 cm), that arose on his back several months prior to presentation. No members of his family have had this condition. Additional history revealed that this patient had no trauma to the area and no history of dermatologic conditions. The patient’s medical history was unremarkable. On examination, the patient was found to have a solitary yellowish, red plaque upon his back, 2 cm by 1 cm in size (Figure 1). No additional plaques or nodules were found anywhere upon the patient. The lesion had not received prior treatment. The remainder of the physical examination was unremarkable. 

 

**Figure 2:**
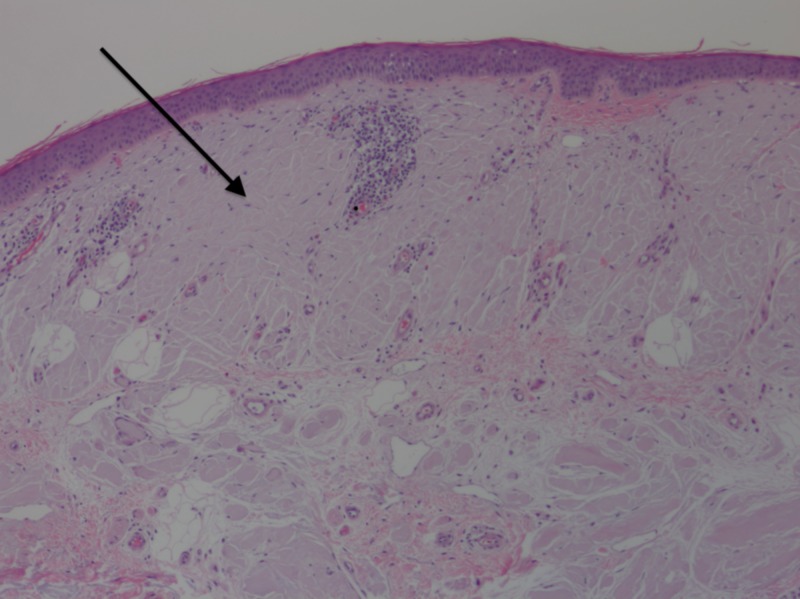
Homogenous amyloid material in the dermis. Arrow indicates amyloid material. Hematoxylin-eosin stain, original magnification 100x.

**Figure 3:**
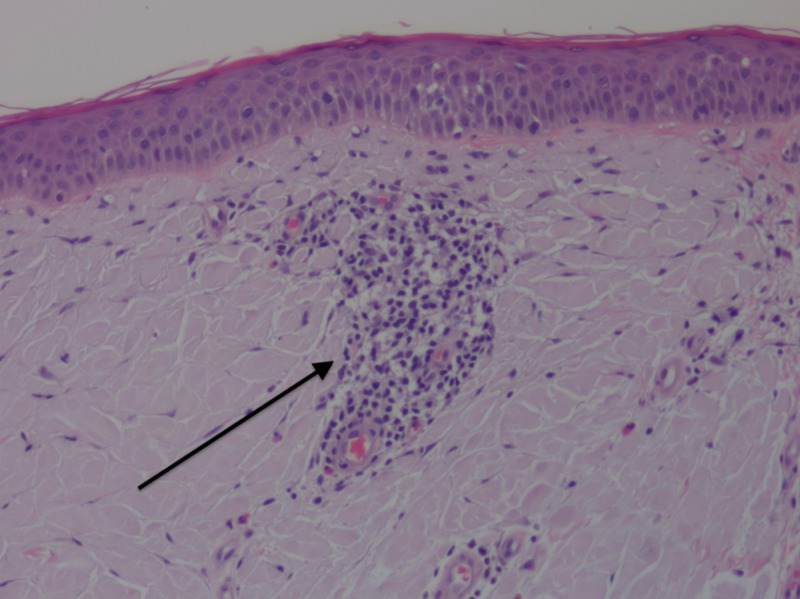
Clusters of perivascular mononuclear cells. Arrow indicates perivascular mononuclear cells. Hematoxylin-eosin stain, original magnification 200x.

**Figure 4:**
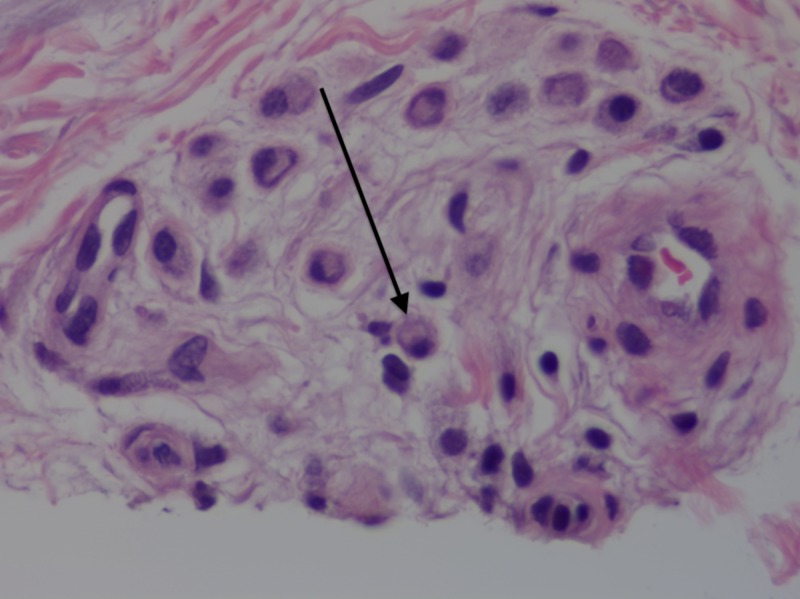
Clusters of perivascular mononuclear cells. Arrow indicates a plasma cell with an eccentric nucleus. Hematoxylin-eosin stain, original magnification 400x.

**Figure 5:**
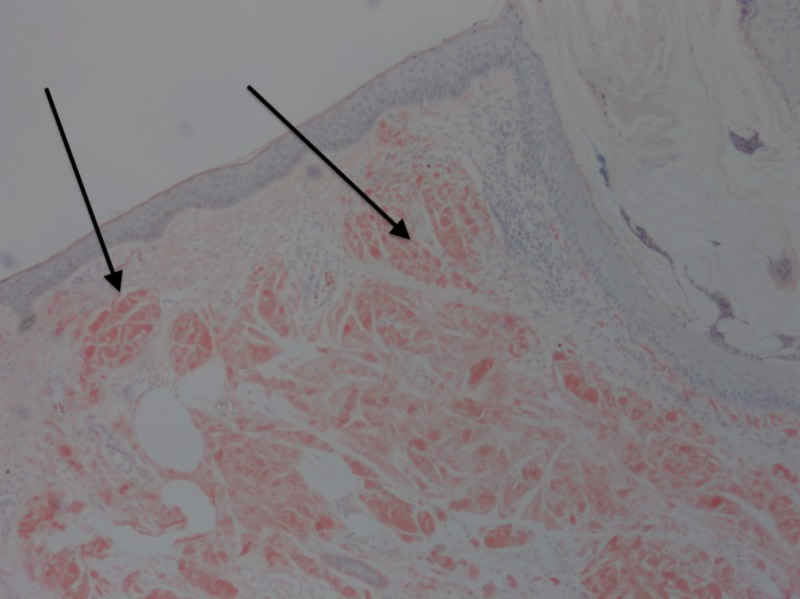
Amyloid material in the dermis. Arrows pointing to amyloid material. Positive Congo Red stain, original magnification 200x.

**Figure 6:**
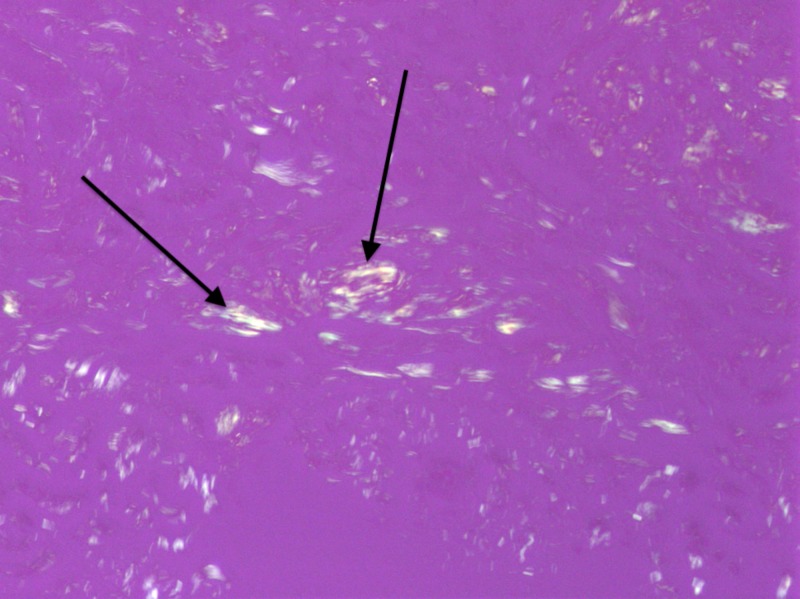
Amyloid deposits birefringent under polarized light. Arrows indicate amyloid deposits. Positive Congo Red stain with polarized light, original magnification 200x.

A shave biopsy was performed, measuring 0.9 by 0.8 cm, and histopathology revealed aggregates of homogenous eosinophilic material throughout the superficial and mid-dermis (Figure 2). Mild perivascular chronic inflammation with eosinophils and aggregates of plasma cells were also seen (Figures 3, 4). A special stain for crystal violet confirmed the material as amyloid. When stained with Congo red and viewed under polarized light, the sample demonstrated amyloid deposits with apple-green birefringence (Figures 5, 6). No keratin was found in the material on special stains with appropriate controls, supporting the extra epidermal origin of the amyloid. Thus, the amyloid deposition was most likely due to immunoglobulins produced either locally or systemically. 

 

 

 

 

 

The patient underwent systemic evaluation, which consisted of comprehensive metabolic panel (Table 1), complete blood cell count (Table 2), serum protein electrophoresis (Table 3), urine protein electrophoresis (Table 4), Sjogren's antibodies (Table 5), rheumatoid arthritis factor (Table 6), and antinuclear antibodies (Table 7). Currently, the patient has a normal renal function, liver function, comprehensive metabolic panel, and complete blood cell count with differential. Serum and urine protein electrophoresis was negative. Sjogren's antibodies, rheumatoid arthritis factor, and antinuclear antibodies were negative. However, the patient was encouraged to undergo longitudinal follow up to monitor for potential progression to systemic disease.

**Table 1:** Comprehensive Metabolic Panel BUN = blood urea nitrogen, eGFR = estimated glomerular filtration rate, AST (SGOT) = aspartate aminotransferase (serum glutamic-oxaloacetic transaminase), ALT (SGPT) = alanine aminotransferase (serum glutamic-pyruvic transaminase)

Comprehensive Metabolic Panel	Result	Units	Reference Interval
Glucose	87	mg/dL	65-99
BUN	19	mg/dL	8-27
Creatinine	1.23	mg/dL	0.76-1.27
eGFR if Non-African American	61	mL/min/1.73	>59
eGFR if African American	70	mL/min/1.73	>59
BUN/Creatinine Ratio	15		10-24
Sodium	141	mmol/L	134-144
Potassium	4.5	mmol/L	3.5-5.2
Chloride	103	mmol/L	96-106
Carbon Dioxide, Total	25	mmol/L	20-29
Calcium	9.3	mg/dL	8.6-10.2
Albumin	4.1	g/dL	3.6-4.8
Globulin, Total	2.9	g/dL	1.5-4.5
Albumin/Globulin Ratio	1.4		1.2-2.2
Bilirubin, Total	0.5	mg/dL	0.0-1.2
Alkaline Phosphatase	63	IU/L	39-117
AST (SGOT)	18	IU/L	0-40
ALT (SGPT)	17	IU/L	0-44

**Table 2:** Complete Blood Count (CBC) with Differential/Platelet WBC = white blood cell count, RBC = red blood cell count, MCV = mean corpuscular volume, MCH = mean corpuscular hemoglobin, MCHC = mean corpuscular hemoglobin concentration, RDW = red cell distribution width

Complete Blood Count (CBC) with Differential/Platelet	Result	Units	Reference Interval
WBC	7.3	X10E3/uL	3.4-10.8
RBC	4.83	X10E3/uL	4.14-5.80
Hemoglobin	15.0	g/dL	13.0-17.7
Hematocrit	43.6	%	37.5-51.0
MCV	90	fL	79-97
MCH	31.1	pg	26.6-33.0
MCHC	34.3	g/dL	31.5-35.7
RDW	13.5	%	12.3-15.4
Platelets	194	X10E3/uL	150-379
Neutrophils	61	%	Not Estab.
Lymphs	22	%	Not Estab.
Monocytes	11	%	Not Estab.
Eosinophils	5	%	Not Estab.
Basophils	1	%	Not Estab.
Neutrophils (Absolute)	4.5	X10E3/uL	1.4-7.0
Lymphocytes (Absolute)	1.6	X10E3/uL	0.7-3.1
Monocytes (Absolute)	0.8	X10E3/uL	0.1-0.9
Eosinophils (Absolute)	0.4	X10E3/uL	0.0-0.4
Basophils (Absolute)	0.1	X10E3/uL	0.0-0.2
Immature Granulocytes	0	%	Not Estab.
Immature Granulocytes (Absolute)	0.0	X10E3/uL	0.0-0.1

**Table 3:** IFE, PE, and FLC, Serum IFE = immunofixation blood test, PE = protein electrophoresis, FLC = quantitative free κ (kappa) and λ (lambda) light chains

IFE, PE, and FLC, Serum	Result	Units	Reference Interval
Immunoglobulin G, Quantitative, Serum	1234	mg/dL	700-1600
Immunoglobulin A, Quantitative, Serum	288	mg/dL	61-437
Immunoglobulin M, Quantitative, Serum	168	mg/dL	20-172
Protein, total	7.0	g/dL	6.0-8.5
Albumin	3.7	g/dL	2.9-4.4
Alpha-1-Globulin	0.1	g/dL	0.0-0.4
Alpha-2-Globulin	0.7	g/dL	0.4-1.0
Beta Globulin	1.1	g/dL	0.7-1.3
Gamma Globulin	1.4	g/dL	0.4-1.8
M (monoclonal) - Spike	Not Observed	g/dL	Not Observed
Globulin, Total	3.3	g/dL	2.2-3.9
Albumin/Globulin Ratio	1.2		0.7-1.7
Free Kappa Light Chains, Serum	17.5	mg/L	3.3-19.4
Free Lambda Light Chains, Serum	23.1	mg/L	5.7-26.3
Kappa/Lambda Ratio, Serum	0.76		0.26-1.65

**Table 4:** PE (Rfx, IFE), Random Urine PE (Rfx, IFE) = protein electrophoresis with interpretation with reflex to IFE

PE (Rfx, IFE), Random Urine	Result	Units	Reference Interval
Protein, Total, Urine	7.9	mg/dL	Not Estab.
Albumin, Urine	38.6	%	
Alpha-1-Globulin, Urine	6.0	%	
Alpha-1-Globulin, Urine	12.3	%	
Beta Globulin, Urine	24.5	%	
Gamma Globulin, Urine	18.4	%	
M (Monoclonal) - Spike, %	Not Observed	%	Not Estab.

**Table 5:** Sjogren’s Antibodies Anti-SS-A = anti-Sjogren's syndrome-related antigen A, anti-SS-B = anti-Sjogren's syndrome-related antigen B, AI = antibody index

Sjogren’s Antibodies, Anti-SS-A/-SS-B	Result	Units	Reference Interval
Sjogren’s Anti-SS-A	<0.2	AI	0.0-0.9
Sjogren’s Anti-SS-B	<0.2	AI	0.0-0.9

**Table 6:** Rheumatoid Arthritis Factor RA = rheumatoid arthritis

Rheumatoid Arthritis Factor	Result	Units	Reference Interval
RA Latex Turbid.	<10.0	IU/mL	0.0-13.9

**Table 7:** Antinuclear Antibodies Direct ANA = antinuclear antibody

Antinuclear Antibodies Direct	Result	Units	Reference Interval
ANA Direct	Negative		Negative

## Discussion

Amyloid is an extracellular protein that appears microscopically as homogenous, amorphous, eosinophilic, and hyaline [1]. Amyloidosis comprises a collection of conditions in which amyloid proteins deposit into tissues of the body. If the amyloid is only deposited within the skin and there is no evidence of systemic involvement, then the condition is termed primary localized cutaneous amyloidosis. However, if the amyloid is systemic and involves several organs or tissues, then it is termed primary or secondary systemic amyloidosis [1].

Within the category of PLCA exists three subcategories: macular, papular, and nodular amyloidosis. Of these three subtypes, the nodular amyloidosis subtype, or PLCA, is the rarest. Gottron was the first to describe nodular amyloidosis. Clinically, PLCA appears as single or multiple nodules that are yellow, brown, or reddish. The nodules are about 0.5 cm to 7 cm in size and are typically present on acral surfaces of the body, trunk, neck, or extremities [2]. Occasionally, multiple nodules may sometimes be found in locations such as the face, scalp, or extremities. Nodular amyloidosis is rarely present on the back, but such is the case in our patient.

The mean age of onset of nodular localized cutaneous amyloidosis is 55 years, with a range of 33 to 86 years [3]. The condition does not show a predilection for men versus women, nor a particular race. Risk factors for the condition have not been established. In most cases, PLCA is benign and limited to the skin. However, it has been noted to progress to systemic disease in about 7% to 50% of patient cases [4]. Also, PLCA lesions may appear histologically similar to lesions found in primary and myeloma-associated systemic amyloidosis [5]. Several case reports have established links between PLCA and Sjogren’s syndrome [6, 7]. In fact, PLCA has been found in 25% of patients with Sjogren’s syndrome [6]. Thus, it has been deemed important for patients to undergo longitudinal follow up to monitor for potential progression to systemic disease. A systemic evaluation consists of complete blood cell count, comprehensive metabolic panel, urinalysis, serum, and urine protein electrophoresis [4].

The pathophysiology of PLCA involves the production of amyloid light chain (AL) fibril proteins by plasma cells. The AL proteins are composed of immunoglobulin light chains, which can also be found in other types of amyloidosis. Immunohistochemical stains have shown the AL proteins to be composed of either kappa or lambda immunoglobulins [8]. The monoclonal plasma cells secrete the AL fibrils in a mechanism that is still unknown, but unique to nodular amyloidosis [9]. The pathophysiology of other subtypes of amyloidosis, such as lichen and macular amyloidosis, involves the secretion of amyloid fibrils by keratinocytes [10].

Because of the extremely characteristic histopathology of amyloidosis, diagnosis is made by biopsy [3]. Histological examination of nodular amyloidosis reveals eosinophilic deposits distributed throughout the dermis. The overlying epidermis may reflect atrophic changes. The walls of small vessels may have amyloid deposits, and perivascular lymphocytes and plasma cells can be observed. This is unique to the nodular variant of amyloidosis. Of note, histopathology is unable to distinguish between localized and systemic amyloidosis. However, an increased amount of plasma cells scattered through the deposits is unique to the nodular amyloidosis subtype [11]. When the sample is stained with Congo red and viewed under polarized light, the amyloid deposits exhibit characteristic apple-green birefringence.

Various treatments for nodular cutaneous amyloidosis exist. The most common method is surgical removal, specifically using a surgical shave technique [3]. In cases of facial nodular amyloidosis, the surgical technique is highly preferred [12]. Other treatment methods include dermabrasion or electrodissection [13, 14]. Laser treatment, specifically with pulsed lasers or carbon dioxide, has also been proved to help patients in some cases [15]. A report of successful treatment with intralesional methotrexate has also been published [16]. Of note, nodular cutaneous amyloidosis lesions may recur even after treatment.

## Conclusions

Nodular amyloidosis is a rare condition that is typically identified on the extremities; however, it can also be located on the lower back. It is important for patients with the condition to undergo a systemic evaluation to monitor for potential progression to systemic disease.
